# Psychiatric comorbidities among patients with complex drug-resistant tuberculosis in Mumbai, India

**DOI:** 10.1371/journal.pone.0263759

**Published:** 2022-02-11

**Authors:** Chinmay Laxmeshwar, Mrinalini Das, Taanya Mathur, Tarun Israni, Santosh Jha, Aparna Iyer, Mabel Morales, Tom Decroo, Tinne Gils, Gabriella Ferlazzo, Kleio Iakovidi, Mariana Garcia, Petros Isaakidis

**Affiliations:** 1 Médecins Sans Frontières, Mumbai, India; 2 Institute of Tropical Medicine, Antwerp, Belgium; 3 Southern Africa Medical Unit, Médecins Sans Frontières, Cape Town, South Africa; The Foundation for Medical Research, INDIA

## Abstract

**Background:**

People with drug-resistant tuberculosis (DR-TB) are known to suffer from many mental-health disorders. This study aims to describe the proportion of patients diagnosed with psychiatric comorbidities, the different psychiatric diagnoses made, and treatment outcomes among DR-TB patients with or without psychiatric comorbidity and initiated on DR-TB treatment between January 2012 and March 2019 at Médecins Sans Frontières independent clinic in Mumbai, India.

**Methods:**

This is a retrospective study using routinely collected clinical data. DR-TB care included individualised treatment, psychosocial support, and integrated psychiatric care.

**Results:**

During the study period, 341 DR-TB patients were enrolled, with a median age of 25 years (IQR:20.0–36.5 years), 185 (54.2%) females, 143 (41.9%) with PreXDR-TB, and 140 (41.0%) with XDR-TB. All 341 patients were screened by a counsellor, 119 (34.9%) were referred for psychiatric evaluation, and 102 (29.9% of 341) were diagnosed with a psychiatric comorbidity. Among 102 diagnosed with a psychiatric comorbidity, 48 (47.0%) were diagnosed at baseline, and 86 (84.3%), or 25.2% of all 341 patients enrolled, were treated with psychotropic drugs. Depressive disorders were diagnosed in 49 (48.0%), mixed anxiety and depression in 24 (23.5%), neurocognitive disorders and anxiety in five (4.9%), and medication induced psychosis in two (2.0%). No anti-TB drugs were significantly associated with psychiatric comorbidities developed during treatment. Of 102 DR-TB patients with a psychiatric comorbidity, 75.5% (77) had successful DR-TB treatment outcomes, compared to 61.1% (146/239) not diagnosed with a psychiatric comorbidity (p = 0.014).

**Conclusion:**

In our setting, among people started on DR-TB treatment, and with a complex TB resistance profile, about one in three patients experienced a psychiatric comorbidity, of which half developed this comorbidity during treatment. With comprehensive psychiatric care integrated into DR-TB care delivery, treatment outcomes were at least as good among those with psychiatric comorbidities compared to those without such comorbidities.

## Background

Tuberculosis (TB) continues to be a leading cause of morbidity and mortality around the world. In 2019, it was estimated that globally TB affected 10 million people and caused 1.4 million deaths [1]. There is also a steady rise in the number of rifampicin-resistant tuberculosis cases (RR-TB) being reported from around the world, and 27% of those were reported from India [1].

Treatment of drug-resistant tuberculosis (DR-TB) is arduous. It includes the use of toxic regimens and exposes people with DR-TB to psychosocial stressors, both of which predispose them to the development of mental health disorders [2]. People with DR-TB must take treatment for at least nine months and may suffer from many adverse events. The regimen includes drugs like cycloserine, fluoroquinolones, and isoniazid that have been associated with psychiatric side effects [3–5]. Of these, cycloserine was reported as the most frequent cause of psychiatric side-effects [5]. People with DR-TB are also exposed to psychosocial stressors like stigma, discrimination, and financial constraints, which have been reported to lead to depression and anxiety [6].

Among the psychiatric comorbidities reported in people treated for DR-TB, depression, anxiety, and psychosis are most common [3, 7–11]. The origin of depression and anxiety is multifactorial. Psychosis is generally reported as an adverse event associated with the use of cycloserine in DR-TB treatment [3, 12]. According to a meta-analysis, the pooled prevalence of depression, anxiety, and psychosis among patients treated for DR-TB were 25% (95% confidence interval (95%CI) 14–39), 24% (95%CI 2–57), and 10% (95%CI 7–14), respectively [12]. However, psychiatric comorbidities among people with DR-TB go beyond depression, anxiety, and psychosis [3, 13].

The diagnosis and management of psychiatric comorbidities in DR-TB patients is difficult for non-specialists [14]. Given the lack of capacity and limited resources to diagnose and manage the entire gamut of psychiatric comorbidities, psychiatric care is usually not integrated with TB care delivery [2, 15], and patients with psychiatric comorbidities are referred to specialists. The integration of adequate psychiatric services within TB programmes is a pillar of the EndTB strategy [16]. However, evidence on how to provide psychiatric care integrated within DR-TB care is scarce.

The humanitarian non-governmental organisation, Médecins Sans Frontières, since 2006 has been providing care to people with DR-TB using a person-centred model, which includes integrated psychiatric services. This study aims to describe the proportion of patients with psychiatric disorders, the different psychiatric diagnoses made, and treatment outcomes among DR-TB patients with or without psychiatric comorbidity and initiated on treatment between January 2012 and March 2019 at a Médecins Sans Frontières independent clinic in Mumbai, India.

## Methods

### Study design

This is a retrospective study using routinely collected clinical data.

### Study setting

The study was conducted in a Médecins Sans Frontières (MSF) independent clinic in Mumbai, India. Mumbai is a metropolis with a population of over 12.5 million [17]. The city is a hotspot for TB reporting 22% of TB cases in the state of Maharashtra [18]. The proportion of drug resistance is also high, with 24% and 41% among new and previously treated patients, respectively, and 34% among TB patients coinfected with the human immunodeficiency virus (HIV) [19, 20].

The MSF clinic has provided free-of-cost care to DR-TB patients since 2007 [21, 22]. The clinic receives patients with complex TB resistance patterns who are referred from public and private healthcare institutions. Most patients coming to the MSF clinic belong to the lower socioeconomic strata. Patients are provided an individualised treatment regimen based on their treatment history and baseline drug-resistance pattern. Since February 2016, bedaquiline and delamanid have been provided to patients with limited treatment options. A description of the determination of resistance patterns and treatment regimens used in the project is available elsewhere [22]. All patients received care from a team of doctors, nurses, counsellors, and a social worker. A consultant psychiatrist was also part of the team.

All patients underwent DR-TB counselling, which followed the motivation-information-behavior skills model. At DR-TB treatment initiation, patients and their primary caregiver received health education addressing the disease and its treatment. This was followed by the preparation of an adherence plan with the patient and their primary caregiver. Social assessment was also conducted, and based on its results, patients were linked to appropriate social support services. Follow-up counselling focused on adherence, identifying adverse events, and any other topics deemed necessary by the counsellor. Counselling consisted of individual and group sessions with the objective of providing emotional, behavioural, and adherence support and providing techniques to manage distress. Peer educators conducted group sessions for all patients with the objective of providing motivation and support by sharing the story of their treatment journey.

The mental health status of all patients was assessed at baseline by a counsellor and every three months after that. The patient health questionnaire– 9 (PHQ-9) was used to evaluate the presence of depressive symptoms, and the alcohol use disorders identification test (AUDIT) questionnaire was used to assess alcohol dependence [23]. Symptoms were also assessed and explored during follow-up counseling sessions and need based assessments were conducted if patients reported any emotional distress. Tailored sessions were conducted for patients with psychiatric comorbidities. Psychological support was provided on-site by trained counselors. Psycho-education sessions were conducted with family members. This helped the caregiver understand the mental status of the patient. Counselors also discussed tips on how caregivers could effectively support the patient.

All patients presenting with signs and symptoms of psychiatric comorbidities were referred, either by a counsellor or by the treating physician, for evaluation to the psychiatrist working within the same clinic. The psychiatrist evaluated these patients and gave a diagnosis when appropriate. While efforts were made to code diagnosis using the Diagnostic and Statistical Manual of Mental Disorders, 5th Edition (DSM-V) criteria, some patient files did not have a diagnosis using DSM-V terminology [24].

### Management of psychiatric comorbidities

All patients diagnosed with psychiatric comorbidities were advised for management with counselling and psychotherapy and, when necessary, were initiated on pharmacological therapy by the psychiatrist. All patients on pharmacological therapy were followed up once a month with the psychiatrist.

Management of depressive disorders included counselling for all and treatment with psychotropic drugs as required. Medicines prescribed included escitalopram (5-10mg/day) or mirtazapine (7.5-45mg/day). These pharmacological agents were preferred owing to the minimal interactions with anti-tuberculosis and HIV medications. Follow-up for patients with these drugs included close monitoring for adverse events. Counselors were responsible for providing emotional support and psycho-education to the patient and their family members. They were also responsible for supporting in the symptomatic management of the mental health issues faced by the patient.

For anxiety disorders, medicines prescribed included short-term treatment with clonazepam (0.25-2mg/day) and lorazepam (0.5-2mg/day). Non-pharmacological interventions included supportive counselling and individualized psychotherapy approaches, including techniques like muscle relaxation, mindfulness, and maintaining a thought diary.

For patients with psychosis, risperidone was the drug of choice. No pharmacological agents were prescribed for neurocognitive disorders.

### Study population

All DR-TB patients enrolled at the MSF clinic in Mumbai between January 2012 till March 2019 were included in the study. All patients had an end of treatment outcome by March 2021.

### Definitions

1. Poly-drug resistant tuberculosis (PDR-TB) was defined as resistance to more than one anti-TB drug, other than isoniazid and rifampicin.
2. Multi-drug resistant tuberculosis (MDR-TB) was defined as resistance to at least isoniazid and rifampicin.
3. Pre extensively drug-resistant tuberculosis (PreXDR-TB) was defined as MDR-TB plus resistance to either a fluoroquinolone or a second line injectables.
4. Extensively drug-resistant tuberculosis (XDR-TB) was defined as MDR-TB plus resistance to fluoroquinolone and second-line injectables.
5. Standard definitions for treatment outcomes (cured, treatment completed, died, treatment failure, lost to follow-up) were used [25]. Cured and treatment completed were categorised as successful treatment outcomes, while died, treatment failure, and lost to follow-up were categorised as unsuccessful treatment outcomes.

### Data collection and analysis

Data were extracted from patient files and the project’s electronic databases. Demographic characteristics (age, sex), clinical characteristics (HIV, TB resistance pattern, site of TB disease, previous treatment, treatment start and end dates, treatment outcome, and regimen), and variables related to the psychiatric care provision (consultation by a counsellor, date of referral for consultation with a psychiatrist, psychiatric diagnosis, and treatment) were collected. Data was exported into SPSS v20 (IBM, NY, USA) for analysis.

Continuous demographic and clinical variables were described using median and interquartile range (IQR), and categorical variables were described using proportions. The association between the presence of psychiatric comorbidities and demographic and clinical variables was assessed using the chi-squared test or Fisher’s exact test based on the number of observations for individual cells. Time to referral for psychiatric evaluation for all conditions was calculated from the initiation of DR-TB treatment.

### Ethics

This research fulfilled the exemption criteria set by the Médecins Sans Frontières Ethics Review Board for a posteriori analyses of routinely collected clinical data and thus did not require MSF ERB review.

## Results

During the study period, 341 DR-TB patients were enrolled at the MSF clinic, with a median age of 25 years (IQR: 20.0–36.5 years) and 185 (54.2%) females. A total of 302 (88.6%) patients were previously treated, and 143 (41.9%) had PreXDR-TB and 140 (41.0%) had XDR-TB. Demographic and clinical characteristics are described in Table 1.

**Table 1:** Demographic and clinical characteristics of drug-resistant tuberculosis patients initiated on treatment at the Médecins Sans Frontières Mumbai clinic between January 2012 and March 2019.

Characteristics		Total n	DR-TB patients diagnosed with psychiatric comorbidity at baseline n (%)	DR-TB patients with diagnosed psychiatric morbidity during treatment n (%)	DR-TB patients without diagnosed psychiatric morbidity n (%)	
**Total**		341	48 (14.1)	54 (15.8)	239 (70.1)	
**Median age, in years (IQR)**		25 (20–36.5)	28.5 (22.7–40.0)	26.0 (20.3–36.0)	25 (19.5–36.0)	
**Sex**						
	Male	156	18 (11.5)	28 (17.9)	110 (70.5)	X^2^ = 2.13 (df = 2) p = .344
	Female	185	30 (16.2)	26 (14.1)	129 (69.7)	
**HIV-coinfection**						
	Seropositive	86	6 (7.0)	14 (16.3)	66 (76.7)	X^2^ = 4.86 (df = 2) p = .880
	Seronegative	255	42 (16.5)	40 (15.7)	173 (67.8)	
**TB site**						
	Pulmonary	286	38 (13.3)	49 (17.1)	199 (69.6)	X^2^ = 2.734 (df = 2) p = .254
	Extra-pulmonary	55	10 (18.2)	5 (9.1)	40 (72.7)	
**Previous treatment**						
	Newly diagnosed	39	8 (20.5)	11 (28.2)	20 (51.3)	X^2^ = 7.773 (df = 2) p = .021
	Previously treated	302	40 (13.2)	43 (14.2)	219 (72.5)	
**TB resistance patterns**						
	PDR-TB	14	1 (7.1)	0 (0.0)	13 (92.9)	NA
	MDR-TB	44	1 (2.3)	5 (11.4)	38 (86.4)	
	PreXDR-TB	143	21 (14.7)	26 (18.2)	96 (67.1)	
	XDR-TB	140	25 (17.9)	23 (16.4)	92 (65.7)	

IQR = Interquartile range; PDR-TB = Polydrug resistant tuberculosis; MDR-TB = Multidrug resistant tuberculosis; PreXDR-TB = pre-extensively drug-resistant tuberculosis; XDR-TB = Extensively drug-resistant tuberculosis; NA = Not Applicable.

### Cascade of psychiatric care

**Fig 1:**
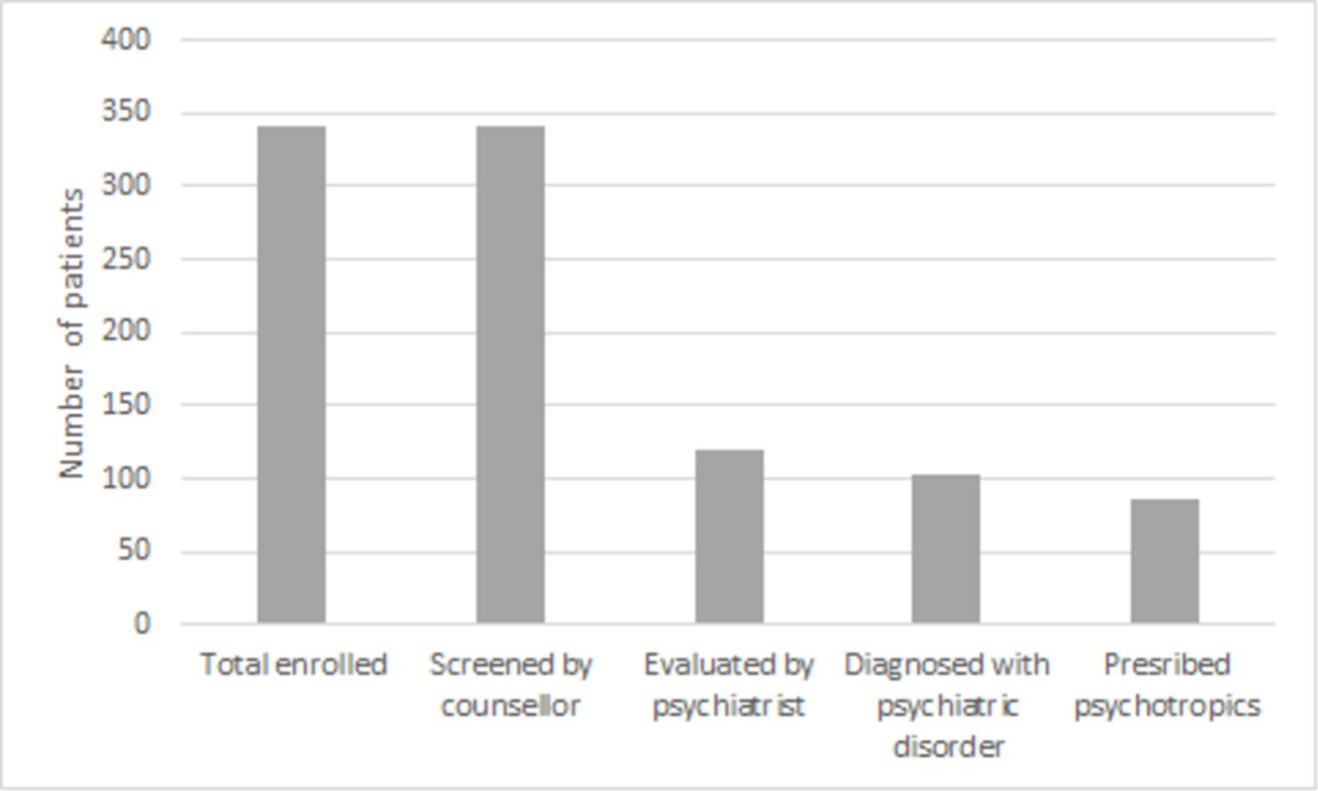
Cascade of psychiatric care among drug-resistant tuberculosis patients enrolled at Médecins Sans Frontières Mumbai clinic between January 2012 and March 2019.

All 341 enrolled patients were screened by a counsellor. Among them, 119 (34.9%) were referred either at baseline or during follow-up to a psychiatrist for evaluation, and 102 (85.7%) were diagnosed with psychiatric comorbidity. Among 102 diagnosed with psychiatric comorbidity, 86 (84.3%), or 25.2% of all 341 patients enrolled, were started on pharmacological therapy. Among 102 diagnosed with psychiatric comorbidities, 48 (47.0%) were diagnosed at baseline. The cascade of psychiatric care is shown in Fig 1.

The main symptoms that led to referral for psychiatric evaluation were the presence of depressive symptoms in 54/116 (46.6%), followed by symptoms of anxiety in 22/116 (19.0%) and behavioural disturbances in 12/116 (12.1%) patients. The median time to referral for a psychiatric evaluation from treatment initiation was 2.6 weeks (range: 0.0–104.0 weeks). For psychosis, the median time to referral from treatment initiation was higher at 19.1 weeks (range: 0.0–71.1 weeks), while for anxiety and depressive symptoms, it was 1.1 weeks (range: 0.0–98.1 weeks). Five (4.3%) patients were referred for reporting suicidal ideation.

Among the 102 diagnosed with psychiatric comorbidity, depressive disorders were diagnosed in 49 (48.0%), with 24 (49.0%) diagnosed at baseline assessment. Twenty-four (23.5%) were diagnosed with mixed anxiety and depression, among whom 13 (54.0%) were diagnosed at baseline. Five (4.9%) each were diagnosed with neurocognitive disorders and anxiety disorders. Two (2.0%) patients were determined to suffer from medication induced psychosis. The complete range of psychiatric diagnosis are shown in Table 2.

**Table 2:** Psychiatric diagnoses among drug-resistant tuberculosis patients enrolled at Médecins Sans Frontières Mumbai clinic between January 2012 and March 2019.

Psychiatric diagnosis	DR-TB patients diagnosed with psychiatric comorbidity at baseline n (%)	DR-TB patients with diagnosed psychiatric morbidity during treatment n (%)	Total
Total	48 (47)	54 (53)	102
Depressive disorders	24 (49)	25 (51)	49
Mixed anxiety and depressive disorder*	13 (54)	11 (46)	24
Neurocognitive disorder	2 (40)	3 (60)	5
Anxiety disorders	2 (40)	3 (60)	5
Unspecified mental disorder*	2 (67)	1 (33)	3
Unspecified behaviour disorders*	2 (100)	0	2
Medication induced psychotic disorder	0	2 (100)	2
Attention deficit hyperactivity disorder	0	2 (100)	2
Sleep wake disorder	0	2(100)	2
Sexual dysfunctions	0	2 (100)	2
Suicide behavior	0	1 (100)	1
Behaviour disorder not amounting to psychosis	0	1 (100)	1
Brief psychotic episode	0	1 (100)	1
Adjustment disorder	1 (100)	0	1
Conversion disorder	1 (100)	0	1
Obsessive compulsive disorder	1 (100)	0	1

* Diagnosis terms do not follow DSM-V.

### Anti-TB drugs and psychiatric comorbidities

Association between anti-TB drugs and psychiatric comorbidities developed during DR-TB treatment is shown in Table 3. No anti-TB drugs were significantly associated with psychiatric comorbidities developed during treatment.

**Table 3:** Association between anti-TB drugs and psychiatric comorbidities among drug-resistant tuberculosis patients enrolled at Médecins Sans Frontières Mumbai clinic between January 2012 and March 2019.

Characteristics		Total n	DRTB patients with diagnosed psychiatric morbidity during treatment n (%)	DRTB patients without diagnosed psychiatric morbidity n (%)	p-value
**On Bedaquiline** (n = 167)					
	Yes	114	26 (22.8)	88 (77.2)	X^2^ = 2.1 (df = 1) p = .147
	No	53	7 (13.2)	46 (86.8)	
**On Delamanid** (n = 157)					
	Yes	133	29 (21.8)	104 (78.2)	X^2^ = 0.32 (df = 1) p = .569
	No	24	4 (16.7)	20 (83.3)	
**On Imipenem**					
(n = 167)	Yes	84	13 (15.5)	71 (84.5)	X^2^ = 1.95 (df = 1) p = .161
	No	83	20 (19.8)	63 (62.4)	
**On Cycloserine** (n = 293)					
	Yes	206	43 (20.9)	163 (79.1)	X^2^ = 2.75 (df = 1) p = .096
	No	87	11 (12.5)	76 (87.5)	
**On Fluoroquinolone** (n = 293)					
	Yes	273	53 (19.4)	220 (80.6)	p = .139*
	No	20	1 (5.0)	19 (95.0)	
**On High dose isoniazid** (n = 284)					
	Yes	11	1 (9.1)	10 (90.9)	p = .696*
	No	273	53 (19.4)	220 (80.6)	

*—Fisher’s exact test.

During management, cycloserine was temporarily withheld for eight patients (out of 13 with cycloserine in their regimen) with major depressive disorder. For one patient, cycloserine had to be withheld intermittently for the entire DR-TB treatment duration. While TB drugs were not considered the cause of anxiety, one patient reported increased anxiety thinking about imipenem infusion, and hence, the infusions were withheld. For two patients with medication induced psychosis, cycloserine was permanently stopped. Cycloserine was temporarily withheld for one patient with a brief psychotic episode.

### TB treatment outcomes

TB treatment outcomes for patients diagnosed with and without psychiatric comorbidities are shown in Table 4. A total of 77 (75.5%) of 102 DR-TB patients with a psychiatric comorbidity were successfully treated, compared to 146 (61.1%) of 239 patients not diagnosed with a psychiatric comorbidity (X^2^ = 6.55; df = 1; p = .014). Of 102 patients with a psychiatric comorbidity, 1 (<1%) was lost to follow-up during treatment, compared to 19 of 239 (7.9%) of patients without psychiatric comorbidity (Fisher Exact test p = .01). Of 48 patients diagnosed with a psychiatric comorbidity at baseline, 32 (66.7%) were treated successfully, compared to 45 of 54 (83.3%) patients diagnosed with a psychiatric comorbidity during treatment (X^2^ = 3.81; df = 1; p = 0.051).

**Table 4:** TB treatment outcomes among drug-resistant tuberculosis patients enrolled at MSF Mumbai clinic between January 2012 and March 2019.

	DR-TB patients diagnosed with psychiatric comorbidity at baseline n (%)	DRTB patients with diagnosed psychiatric morbidity during treatment n (%)	DRTB patients without diagnosed psychiatric morbidity n (%)
**Outcomes**			
Cured	22 (45.8)	36 (66.7)	101 (42.3)
Completed	10 (20.8)	9 (16.7)	45 (18.8)
Died	9 (18.8)	7 (13.0)	60 (25.2)
Failed	6 (12.5)	2 (3.6)	14 (5.8)
Lost to follow-up	1 (2.1)	0	19 (7.9)
**Treatment success**			
Successful outcome	32 (66.7)	45 (83.3)	146 (61.1)
Unsuccessful outcome	16 (33.3)	9(16.7)	93 (38.9)
Total	48	54	239

## Discussion

Among 341 patients initiated on TB treatment and with complex resistance patterns, almost one in three (30%) were diagnosed with a psychiatric comorbidity, of which half were diagnosed at baseline, and about half were diagnosed during follow-up. One in four of 341 patients were treated with psychotropic drugs. The proportion of patients diagnosed at baseline and during treatment is similar to that reported from other cohorts [3]. However, most previous studies included patients on treatment with drug-sensitive TB or MDR-TB, while here, we report on a cohort of patients with complex TB resistance patterns, many on a regimen with new TB drugs, and previously exposed for a long duration to TB treatment [22].

We observed a significantly higher proportion of psychiatric comorbidities among newly diagnosed people with DR-TB than those previously treated. While the proportion of newly diagnosed patients is low in our cohort, we believe that the psychological stress of being diagnosed with a resistant form of TB, which is well known to be associated with having a poor treatment outcome, might have precipitated this. Previous studies have reported that psychosocial stress can trigger mental health illnesses in people with DR-TB, which might be more prominent among those newly diagnosed [12].

Given the stigma associated with psychiatric illnesses and TB, patients might not be inclined to report any psychiatric symptoms. The TB clinician or counsellor is best placed to overcome these barriers and identify those in need of psychiatric care. When systematic screening is not integrated as part of to be provided TB services, psychiatric comorbidities might be missed [2].

Among those diagnosed with psychiatric comorbidities, depressive disorders and mixed anxiety and depression were most common. Previous studies have also reported a high proportion of people with DR-TB suffering from depression and anxiety [3, 10, 12, 26, 27]. These conditions were almost equally diagnosed at baseline and during treatment. Along with depression and anxiety, a broad spectrum of psychiatric comorbidities were diagnosed among our cohort. Management of psychiatric comorbidities required pharmacological therapy along with psychological counselling. Diagnosis and management of all these comorbidities were possible due to the model of care with systematic counselling and the availability of a psychiatrist within the same health facility where DR-TB care was provided.

In our cohort, patients with psychiatric comorbidities did not have poorer DR-TB treatment outcomes compared to patients not diagnosed with any psychiatric comorbidity. Very few were lost to follow-up, even less than among those without a psychiatric comorbidity. We speculate that with the inclusion of comprehensive, integrated psychiatric care, people with DR-TB can successfully complete their treatment. The provision of person-centered care may have motivated patients with a psychiatric comorbidity to stay in care. A study from Peru also reported no association between psychiatric conditions and having an unfavourable outcome among XDR-TB patients [28]. The inclusion of mental health care in TB programmes has been recommended by many studies [2, 7, 8, 12, 13, 29]. However, most programmes include only health education and treatment literacy, while there is a lack of psychological counselling and the availability of a specialist to manage patients who might require pharmacological interventions [2].

The strength of this study lies in the detailed description of psychiatric comorbidities among DR-TB patients at baseline and during treatment, which is often missed in routine programmes. This study includes patients from a specialised treatment centre for patients with complex resistance patterns and with frequent use of new drugs, which may affect the generalisability of our findings. However, we believe that the model of care described here can be adapted and serve as a guide while designing mental health support among programmes providing care to people with DR-TB. Another limitation is the use of retrospective programme data for this study. Even though unfavorable outcomes were statistically less frequent among those with a psychiatric comorbidity, our study design did not allow to assess causality. Selection bias may have occurred in the subgroup diagnosed with a psychiatric comorbidity during treatment (thus among those retained in care). Still, we are confident to conclude that outcomes were at least as good among those with psychiatric comorbidity, given the outcomes obtained among those with a psychiatric comorbidity diagnosed before starting treatment. The retrospective design also limited the number of variables related to mental health that we could extract, including non-DSM-V diagnosis terms for some patients. Data on outcomes for psychiatric care were not collected.

## Conclusion

In our setting, among people started on DR-TB treatment and with a complex TB resistance profile, about one in every three patients experienced a psychiatric comorbidity, of which half developed this psychiatric comorbidity during treatment. Systematic mental health screening and involvement of a psychiatrist were key to diagnosing a wide spectrum of psychiatric conditions. With comprehensive psychiatric care integrated into DR-TB care delivery treatment outcomes were at least as good among those with psychiatric comorbidities compared to those without such comorbidities.
