# Correction: Psychiatric comorbidities among patients with complex drug-resistant tuberculosis in Mumbai, India

**DOI:** 10.1371/journal.pone.0298137

**Published:** 2024-01-29

**Authors:** Chinmay Laxmeshwar, Mrinalini Das, Taanya Mathur, Tarun Israni, Santosh Jha, Aparna Iyer, Mabel Morales, Tom Decroo, Tinne Gils, Gabriella Ferlazzo, Kleio Iakovidi, Mariana Garcia, Petros Isaakidis

There are errors in the Funding and Competing Interests statements. The correct statements are:

**Funding:** MSF provided support for this study in the form of salaries for CL, MD, TM, TI, SJ, AI, MM, GF, KI, MG, and PI, and in the form of the publication fees. The specific roles of these authors are articulated in the ‘author contributions’ section.

**Competing Interests**: The authors have read the journal’s policy and have the following competing interests to declare: MSF provided support for this study in the form of salaries for CL, MD, TM, TI, SJ, AI, MM, GF, KI, MG, and PI. This does not alter our adherence to *PLOS ONE* policies on sharing data and materials. There are no patents, products in development or marketed products associated with this research to declare.
